# Genetic diversity at major histocompatibility complex and its effect on production and immune traits in indigenous chicken breeds of India

**DOI:** 10.5194/aab-63-173-2020

**Published:** 2020-06-16

**Authors:** Santosh Haunshi, Divya Devara, Kannaki Ramasamy, Rajkumar Ullengala, Rudra Nath Chatterjee

**Affiliations:** ICAR-Directorate of Poultry Research, Rajendranagar, Hyderabad 500030, India

## Abstract

The genetic diversity at major histocompatibility complex (MHC) in indigenous chicken breeds of India (Ghagus and Nicobari) in comparison with the White Leghorn (WLH) breed was investigated by genotyping the MHC-linked LEI0258 marker. Altogether 38 alleles and 96 genotypes were observed among three breeds. The observed and effective alleles were highest in Ghagus (23, 8.3) followed by Nicobari (14, 3.2) and WLH (10 and 2.2) breeds. The size of alleles ranged from 193 to 489 bp in Ghagus, 193 to 552 bp in Nicobari and 241 to 565 bp in the WLH breed. The number of private alleles was also highest in Ghagus (18) followed by Nicobari (8) and WLH (5) breeds. The most frequent allele was 261 bp in WLH (66 %), 343 bp in Nicobari (50.4 %) and 309 bp in the Ghagus (28.15 %) breed. Observed and expected heterozygosities were highest in Ghagus (0.83, 0.88) followed by Nicobari (0.58, 0.68) and WLH (0.53, 0.54). The genetic distance (Nei) between Ghagus and Nicobari breeds (2.24) was higher as compared to that of Ghagus and WLH (1.23) and that between Nicobari and WLH breeds (0.89). Association analysis revealed significant influence of MHC alleles on body weight, egg production in Ghagus and WLH breeds and antibody titres to Newcastle disease vaccine in the Nicobari breed.

## 1 Introduction

Slow-growing indigenous chickens possess unique attributes like attractive multicoloured plumage, hardiness, an ability to adapt to low input suboptimal rearing conditions and survive under harsh environments, broodiness, perceived desirable taste and flavour of meat and eggs, etc. Rearing of indigenous chickens generates subsidiary income by utilizing minimum inputs and minimum human attention. It also helps in gender empowerment and social upliftment of the rural/tribal people as mostly women and children are involved in rearing of indigenous chickens besides providing household nutritional security. Further, they cater to the needs of consumers for coloured birds and light-brown-shelled eggs in the niche market. Indigenous chickens are reported to be resistant to bacterial, protozoal, fungal and parasitic diseases as they were subjected to many years of natural selection under free-range or backyard systems of rearing (Besbes, 2009). Previous studies have reported the differences in immune competence traits between indigenous and improved chicken breeds owing to different genetic backgrounds. Nicobari, a slow-growing indigenous breed was reported to be relatively resistant to viral diseases like Newcastle disease, Marek's disease (Rai and Ahlawat, 1995) and infectious bursal disease (Sunder et al., 2004; Chatterjee and Yadav, 2008). Furthermore, expression profiling of various pattern recognition receptor genes in indigenous and White Leghorn (WLH) birds revealed the highest expression of *TLR1LB, MDA5, LGP2, B-Lec, IL1* β and *IL8* genes in Ghagus in contrast to WLH birds (Haunshi et al., 2017). These receptor genes are known to play a significant role in innate immune competence. In another study, it was demonstrated that expression of the *TLR4* gene in lipopolysaccharide treated peripheral blood mononuclear cells was significantly higher in indigenous chicken breeds (Aseel and Ghagus) when compared to improved (Dahlem Red and Broilers) chickens (Karnati et al., 2015). Similarly, the expression of *TLR4, TLR5* and *TLR7* genes was significantly higher in indigenous breeds (Kadaknath and Aseel) than those of the WLH breed (Kannaki et al., 2010). General immune competence as assessed by cutaneous basophil hypersensitivity response against PHA-P and cytotoxic T lymphocytes (CD8+) count was also found to be higher in indigenous chicken breeds compared to the Dahlem Red breed (Yadav et al., 2018). Recently in our challenge study, it was observed that the Nicobari breed was relatively resistant to fowl typhoid (*Pastorella multocida*) infection as mortality of the Nicobari breed was remarkably lesser as compared to the improved Vanaraja cross (Kannaki Ramasamy et al., unpublished data). Therefore, it is evident that the genetic background of the chicken is known to influence the disease resistance and immune competence traits (Emam et al., 2014).

Major histocompatibility complex (MHC) in chickens is known to play a significant role in disease resistance, immune competence and production traits (Miller and Taylor, 2016) including resistance to ectoparasites like northern fowl mites (Owen et al., 2008). Associations between the haplotypes of the MHC-B locus and responses to Rous sarcoma virus (Suzuki et al., 2010) and resistance to avian coronavirus (Banat et al., 2013) were also demonstrated. MHC-linked LEI0258 alleles were reported to be significantly associated with production traits in WLH (Lunden et al., 1993) and indigenous Khorasan chickens of Iran (Nikbakht and Esmailnejad, 2015). Recently, it was shown that LEI0258 alleles were associated with humoral and cell mediated immune response in broiler chickens (Esmailnejad et al., 2017). Alleles of the LEI0258 VNTR marker are associated with the serologically defined MHC B haplotypes (Fulton et al., 2006). Various studies have used genotyping of the LEI0258 marker to assess the genetic diversity at MHC (Nikbakht et al., 2013; Han et al., 2013; Guangxin et al., 2014). In a study, Baelmans et al. (2005) detected completely unknown haplotypes using serological method in four lines of indigenous chickens of India, thus reflecting the potentially interesting genetic pool at MHC. Therefore, the present study was carried out with the objective of investigating the genetic variability at MHC in indigenous and WLH breeds of chicken and to study the association of alleles of LEI0258 on growth, production, immunity and survivability traits in indigenous chicken breeds in comparison with the improved WLH breed.

## 2 Materials and methods

### 2.1 Experimental birds

A total of 363 birds from three breeds of chicken were used for the present study. Ghagus and Nicobari were the indigenous breeds of India, while a control population of White Leghorn (WLH) was the improved chicken breed. Breeding stocks of all three breeds were kept at the institute farm. Ghagus is phenotypically characterized by brown to red-coloured plumage, pea comb, white skin and brown eyes with high incidence of broodiness behaviour. Nicobari is small bird with brownish matt-coloured plumage and short legs that originated in the Andaman and Nicobar islands of India (Ahlawat et al., 2004). All three breeds were maintained as random bred closed populations. Chicks from three breeds were hatched simultaneously and reared on a floor (deep litter) up to 20 weeks of age, then in individual cages from 21 to 72 weeks of age in an open-sided house. The details of rearing and management, healthcare and vaccinations of birds were described previously (Haunshi et al., 2019).

### 2.2 Blood sampling and DNA isolation

At 20 weeks of age, blood samples from female birds of 119 Ghagus, 125 Nicobari and 119 WLH were collected from a wing vein of birds in 0.5 M EDTA anticoagulant, thoroughly mixed and stored at -20 ${}^{\circ }$C till further use. Genomic DNA from whole blood was extracted using a phenol-chloroform-isoamyl alcohol method (Sambrook and Russell, 2001). Briefly, about 50 µL of whole blood was mixed with lysis buffer and proteinase-K and vortexed. The mixture was incubated in a water bath at 37 ${}^{\circ }$C for 12 h. Equal quantity of phenol was added to the incubated mixture, gently mixed and centrifuged at 12000g for 10 min. Supernatant was transferred to a fresh tube. Further extraction was done with phenol : chloroform : isoamyl alcohol (25 : 24 : 1) and chloroform : isoamyl alcohol (24 : 1). Finally, aqueous phase was transferred to a fresh tube having 3 M sodium acetate and two volumes of chilled isopropanol and mixed gently. The pelleted DNA was dissolved in nuclease-free water. Quality and quantity of DNA was checked with the NanoDrop spectrophotometer. The present experiment was approved by the Institutional Animal Ethics Committee (IEAC).

### 2.3 Genotyping by PCR

Alleles of the LEI0258 marker were identified through PCR. The nucleotide sequences of primers used for amplification of the LEI0258 marker were CACGCAGCAGAACTTGGTAAGG forward and AGCTGTGCTCAGTCCTCAGTGC reverse (McConnell et al., 1999). Forward primer to amplify the LEI0258 marker through PCR was labelled with 6-FAM dye. PCR was carried out using 0.13 µM each for forward and reverse primers, 1.5 mM ${\mathrm{MgCl}}_{2}$, 0.6 units of Dream Taq DNA polymerase (Thermo Fisher Scientific) 200 µM of dNTPs, and 50 ng of template (genomic DNA) in a final reaction volume of 25 µL in a thermal cycler (Veriti, Applied Biosystems, Foster City, USA). Amplification conditions used were initial denaturation at 95 ${}^{\circ }$C for 10 min followed by 40 cycles of 95 ${}^{\circ }$C for 20 s, 62 ${}^{\circ }$C for 20 s and 72 ${}^{\circ }$C for 20 s, followed by a final extension of 72 ${}^{\circ }$C for 10 min. Initially, PCR products were resolved on 3.0 % agarose gel in 1 X TBE buffer at 80 V for 1.5 h to determine the approximate size of alleles. Subsequently, the left-over PCR product was mixed with Hi-Di Formamide and LIZ-500 marker, denatured, and run on an ABI-3500 genetic analyser (Chromous Biotech, Bengaluru). Fragment analysis was performed using the GeneMapper software to determine the size of alleles of the LEI0258 marker precisely.

### 2.4 Sequencing

The exact size of alleles (19 alleles which were predominantly observed among three breeds) of LEI0258 was determined by sequencing the PCR products obtained using primers that bind just outside of the LEI0258 marker. The primers used to acquire entire region of the LEI0258 marker were TCGGGAAAAGATCTGAGTCATTG forward (CAJF01F) and TGATTTTCAGATCGCGTTCCTC (CAJF01R) for the reverse direction (Fulton et al., 2006). The PCR was performed in a final volume of 25 µL with 50 ng of chicken genomic DNA, 2.5 µL of buffer (10×), 0.67 µL of each forward and reverse primers, 0.5 µL of 10 mM dNTP, 0.6 U of Taq DNA polymerase, and water. The PCR cycles included 95 ${}^{\circ }$C for 10 min, 40 cycles of 95 ${}^{\circ }$C for 20 s, 62 ${}^{\circ }$C for 20 s, 72 ${}^{\circ }$C for 20 s and 72 ${}^{\circ }$C for 10 min. The PCR products were separated on agarose gel, and desired fragments were gel eluted using a QIAquick gel extraction kit (Qiagen GmbH, Hilden, Germany) following manufacturer's instructions. These eluted products were sequenced in both directions using BigDye terminator sequencing on an ABI 3730 sequencer (Bioserve Biotechnologies Pvt. Ltd., Hyderabad, India). Sequences of both forward and reverse directions were aligned using BioEdit software to determine the consensus sequence of alleles. ClustalW functions of the BioEdit software were used to investigate the SNP and indel polymorphisms in the flanking regions of R12 and R13 repeats.

### 2.5 Population genetic analysis

The alleles were identified by their sizes. The observed ($N_{a}$) and effective ($N_{e}$) allele number, allele frequency, expected, unbiased expected, and observed heterozygosities ($H_{e}$, $uH_{e}$ and $H_{o}$, respectively) for the LEI0258 locus were estimated using GenAlEx software version 6.502 (Peakall and Smouse, 2006, 2012). The amount of gene diversity was measured by the number of alleles and the unbiased expected heterozygosity, according to the formula proposed by Nei (1973). Genetic identity and genetic distance among the three breeds were calculated from allele frequency according to Nei's unbiased estimates (1978). Deviation from Hardy–Weinberg equilibrium (HWE) was estimated using the analysis of molecular variance (AMOVA) function of GenAlEx software.

### 2.6 Association analysis

The data on growth traits such as body weight and shank length at 4, 8, 16, and 40 weeks of age, production traits such as average age at first egg, egg production up to 72 weeks of age, immunity traits like antibody titres to Newcastle disease vaccine (NDV), and natural antibody (NAb) titres to rabbit RBC (RRBC) at 20 weeks of age recorded in the three breeds previously (Haunshi et al., 2019) were used for the association study. The association of LEI0258 alleles with various traits was determined using following regression model

1 $$Y_{i}=\mu +\Sigma b_{j}f_{ij}+\varepsilon_{i},$$

where, $Y_{i}$ is a dependent variable for specific trait in ith chicken; μ is a general mean; $f_{ij}$ is the copy number of the jth allele of LEI0258 in the ith chicken; $b_{j}$ is half the substitution effect of the jth allele; and $\varepsilon_{i}$ is the residual effect (Esmailnejad et al., 2017). For each allele, all hens were considered as either carrier (1) or non-carrier (0), and then coefficient effect of each allele was determined in comparison to the reference allele. The most frequent allele in each breed was designated as the reference allele, and the association was carried out using regression analysis (SPSS Ver. 12).

## 3 Results

A total of 19 alleles (Table 1) were sequenced in bi-direction and aligned, and consensus size was determined. It was observed that the sizes of alleles detected by capillary electrophoresis (fragment lengths) and sequencing did not match. Overall the difference in size ranged from 1 to 20 bp. There was a pattern in the size difference. The smaller the size of alleles, the smaller the difference in size was. The size difference of 1 bp was observed for 195, 262 and 296 bp alleles. Likewise, the size differences were found to be 3 bp for 312 and 346 bp alleles, 4 bp for 361, 373 and 385 bp alleles, 6 bp for 451 and 495 bp alleles, 13 bp for 552 bp allele, and finally 20 bp for 572 and 585 bp alleles. This pattern of difference in size was used for extrapolating the size of remaining genotyped alleles (Fulton et al., 2006). Similar to the findings of the present study, a size difference of 2 to 17 bp was reported by Mwambene et al. (2019). However, Han et al. (2013) reported a higher (1 to 65 bp) difference in the size of genotyped allele and that determined by the sequencing.

**Table 1:** Polymorphism in LEI0258 alleles in Ghagus, Nicobari and White Leghorn breeds.

Breed	Genotyped allele size,	Sequenced allele size,	Accession	Upstream		R13	R12	Downstream				% identity	Reference number
	bp	bp	No.	-30 to -29	-28	(1 to 29)	(3 to 27)	11 to 18	+26	+28	+34		
				TT/Δ	G/A	n	n	ATTTTGAG/Δ	A/T	Δ/A	A/T		
Nicobari	191	193	MT291853	TT	G	1	3	Δ	A	Δ	T	100	MG991239.1
Ghagus	195	194	MT291851	TT	G	1	3	Δ	A	A	T	99.0	DQ239561.1
WLH	262	261	MT291858	TT	G	1	8	ATTTTGAG	A	Δ	A	100	MG991228.1
WLH	262	261	MT291859	TT	G	1	8	ATTTTGAG	A	Δ	A	100	MG991228.1
Nicobari	261	261	MT291855	TT	G	1	8	ATTTTGAG	A	Δ	A	100	MG991228.1
Nicobari	296	295	MT291854	Δ	G	1	11	ATTTTGAG	A	Δ	T	100	MG991230.1
Ghagus	312	309	MT291847	TT	G	1	12	ATTTTGAG	A	Δ	T	100	MG991138.1
Nicobari	346	343	MT291857	Δ	A	1	15	ATTTTGAG	A	Δ	T	100	KF535098.1
Ghagus	361	357	MT291848	TT	G	1	16	ATTTTGAG	T	Δ	T	100	MG991233.1
Nicobari	373	369	MT291852	TT	G	1	17	ATTTTGAG	A	Δ	A	100	MG991229.1
WLH	385	381	MT291862	TT	G	1	18	ATTTTGAG	T	Δ	T	100	MG892323.1
WLH	407	403	MT291860	Δ	A	1	20	ATTTTGAG	A	Δ	T	99.0	MG892288.1
Ghagus	451	445	MT291846	TT	G	17	6	ATTTTGAG	A	Δ	T	98.6	MG518250.1
Ghagus	495	489	MT291849	TT	G	1	27	ATTTTGAG	A	Δ	A	100	KF535106.1
Ghagus	494	489	MT291850	TT	G	1	27	ATTTTGAG	A	Δ	A	100	KF535106.1
WLH	552	539	MT291864	TT	G	27	3	ATTTTGAG	A	Δ	T	99.6	DQ239516.1
WLH	567	552	MT291861	TT	G	28	3	ATTTTGAG	A	Δ	T	100	MG518313.1
Nicobari	572	552	MT291856	TT	G	28	3	ATTTTGAG	A	Δ	T	100	MG518313.1
WLH	585	565	MT291863	TT	G	29	3	ATTTTGAG	A	Δ	T	97.7	MG518313.1

Previous studies have reported two types of polymorphisms in the LEI0258 marker. One is the number of repeats of the R13 and R12 motifs, and the other is the number of SNPs and indels upstream (-78, -72 or -62 bp) and downstream (88, 67 or 51 bp) of these conserved repeat motifs (Fulton et al., 2006; Guangxin et al., 2014; Chazara et al., 2013). The number of 13 bp (CTATGTCTTCTTT) repeat (R13) varied from 1 to 29 times, while that of 12 bp (CTTTCCTTCTTT) repeat (R12) varied from 3 to 27 times. We have observed one deletion (the TT indel at position -30 to -29 bp, i.e. 29 bp before the R13 repeats) and one SNP (G/A, -28) in an upstream position (77 bp). Two indels (ATTTTGAG at +11 to +18 bp and /A at +28 bp) and two SNPs (A/T, +26 and A/T, +34) were identified downstream (68 bp) of R12 repeats. The position of indel marker “ATTTTGAG” (+11 to +18 bp downstream of last R12 repeat) is different from that reported previously (Fulton et al., 2006; Han et al., 2013; Chazara et al., 2013). A conserved 8 bp sequence, “CTTTCCTT”, was observed after the final R12 repeat in all the sequenced samples. BLASTting of all 19 sequenced alleles in the NCBI nucleotide database indicated that 14 alleles are identical (100 %) to their corresponding accessions from the GenBank database. However, similarities of five sequences against their corresponding accessions in GenBank were slightly less than 100 % (Table 1). All the 19 sequences submitted to NCBI GenBank were provided with accession numbers which ranged from MT291846 to MT291864.

**Figure 1:**
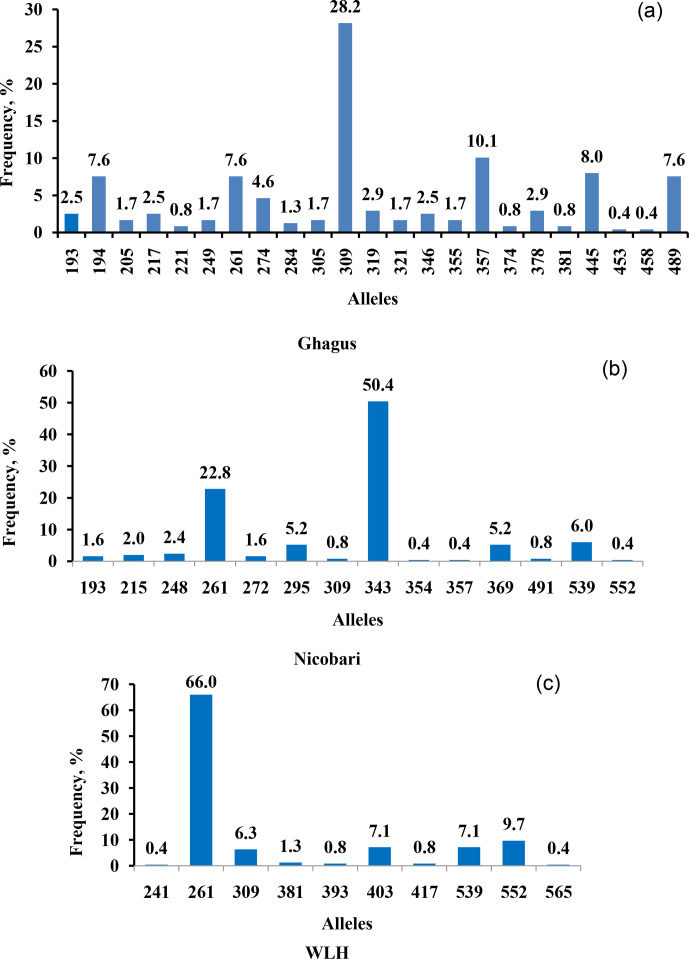
Allele frequency of LEI0258 locus in **(a)** Ghagus, **(b)** Nicobari and **(c)** WLH breeds.

Genetic diversity at major histocompatibility (MHC) in Ghagus, Nicobari and WLH breeds was studied by genotyping the MHC-linked LEI0258 marker. Overall 38 alleles with size ranging from 193 to 565 bp and 96 genotypes were observed in three breeds (Fig. 1). In Ghagus, a total of 23 alleles and 52 genotypes were found. The size of alleles ranged from 193 to 489 bp. The most frequent allele was 309 bp (28.15 %), and the least frequent alleles were 453 and 458 bp (0.42 %). The most frequent genotypes occurring were 309/309, 309/357 and 309/445 bp, each with the proportion of 6.72 %. A total of 14 alleles and 27 genotypes were observed in Nicobari with allele size varying from 193 to 552 bp. In this breed the most and least frequent alleles were 343 bp (50.4 %) and 354, 357 and 552 bp (0.40 %), respectively. The most frequent genotypes were on the order of 343/343 (29.6 %), 261/343 (19.2 %) and 261/261 bp (8.0 %). In WLH, a total of 10 alleles were seen with the most and least frequent ones being 261 bp (66 %) and 565 bp (0.42 %), respectively. The allele size varied from 241 to 565 bp, and among the 17 observed genotypes, 261/261 (47.9 %), 261/403 (9.2 %) and 261/552 bp (8.4 %) genotypes were the most frequently occurring ones.

Number of genotypes, number of observed alleles ($N_{a}$) and effective alleles ($N_{e}$) were highest in Ghagus followed by Nicobari and WLH breeds (Table 1). Similarly, the number of private alleles was highest in the Ghagus breed (78.3 % of total 23 alleles). The next-highest number of private alleles was observed in Nicobari (57.1 % of total 14 alleles) followed by the WLH (50 % of total 10 alleles) breed. Private alleles are those alleles which occurred only in a particular breed and not shared by other breeds, at least in this study.

Expected and unbiased expected heterozygosities were highest in Ghagus followed by Nicobari and WLH breeds (Table 2). As a result, significant (P>0.0001) difference in heterozygosity among the three breeds was observed (Table 3). Heterozygosity was high in Ghagus and Nicobari breeds with a proportion of 84 % and 57.6 % heterozygous genotypes, respectively, as compared to 49.6 % of heterozygous genotypes in the WLH breed. Based on observed and expected genotype frequencies, it was observed that the WLH breed did not deviate from Hardy–Weinberg Equilibrium (HWE) (p=0.331), whereas Ghagus and Nicobari breeds significantly (p<0.0001) deviated from HWE. The genetic distance (Nei) between Ghagus and Nicobari breeds was higher as compared to that of Ghagus and WLH and that between Nicobari and WLH breeds (Table 4).

**Table 2:** Effective number of alleles, heterozygosity and fixation index in three chicken breeds.

Breeds	$N_{a}$	$N_{e}$	$N_{\mathrm{pa}}$	$H_{o}$	$H_{e}$	$uH_{e}$	F
Ghagus	23.0	8.3	18	0.83	0.88	0.88	0.05
Nicobari	14.0	3.2	8	0.58	0.68	0.69	0.16
WLH	10.0	2.2	5	0.53	0.54	0.54	0.02
Mean ± SE	15.7±3.80	4.5±1.9		0.65±0.09	0.70±0.09	0.70±0.10	0.08±0.04

$N_{a}$: observed number of alleles; $N_{e}$: effective number of alleles; $N_{\mathrm{pa}}$: number of private alleles; $H_{o}$: observed heterozygosity; $H_{e}$: expected heterozygosity; $uH_{e}$: unbiased expected heterozygosity; F: fixation index.

**Table 3:** Percentage of homozygous and heterozygous individuals among three breeds.

Breed	Ghagus		Nicobari		WLH	
Status	No.	%	No.	%	No.	%
Heterozygous	100	84.03	72	57.60	59	49.58
Homozygous	19	15.97	53	42.40	60	50.42
Total	119	100	125	100	119	100

χ value 33.58; P>0.0001.

**Table 4:** Genetic identity/distance among three breeds of chicken.

Population/breed	Ghagus	Nicobari	WLH
Ghagus	–	0.106	0.293
Nicobari	2.244	–	0.411
WLH	1.227	0.889	–

Genetic identity (above the diagonal) and genetic distance (below the diagonal) were calculated from allele frequency according to Nei's (1978) unbiased estimates.

Significant association was observed between alleles of the LEI0258 marker and body weight in the Ghagus breed (Table 5). As compared to the reference (309 bp) allele, the 194 bp allele had a significant positive influence on body weight recorded at 4 and 8 weeks of age. The 194 bp allele also had a significant positive influence on shank length at 8 weeks of age, egg production and survivability up to 72 weeks of age. However, the 445 bp allele had a significant negative effect on body weight recorded at 40 weeks of age. This allele also had a nonsignificant negative influence on body weight recorded at other ages. None of the alleles had a significant effect on age at first egg, shank length at 16 weeks of age and immunity traits measured at 20 weeks of age in this breed.

**Table 5:** Association of LEI0258 alleles with survivability, production and antibody traits in indigenous and WLH breeds of chicken.

Traits	LEI0258 allele, bp	Allele effect	SE	P value
Ghagus				
Body weight at 4 weeks	194 (BW3)	34.8	14.5	0.025
Body weight at 8 weeks	194	100.7	42.4	0.030
Body weight at 16 weeks	194	111.3	62.8	NS
Shank length at 8 weeks	194	7.59	2.33	0.003
Egg production up to 72 weeks	194	60.9	29.6	0.05
Survivability up to 72 weeks	194	93.7	39.3	0.048
Body weight at 40 weeks	445	-263.2	86.4	0.003
Nicobari				
SpAb titre to NDV at 20 weeks of age	261 (B15 or B2)	0.99	0.43	0.023
	295 (B5)	0.58	0.26	0.026
WLH				
Body weight at 16 weeks	403	-104	42.4	0.016
Egg production up to 72 weeks	309 (B24)	-81.1	27.3	0.016
	552 (B19.1)	-48.7	19.3	0.013
Survivability up to 72 weeks	309	-53.2	20.4	0.011

Estimates of allele effects relative to 309 bp (reference allele) in Ghagus, 343 bp (reference allele) in Nicobari and 261 bp (reference allele) in the WLH breed based on formula $Y_{i}=\mu +\Sigma b_{j}f_{ij}+\varepsilon_{i}$. SE: standard error; NS: not significant.

In the Nicobari breed, out of 16 alleles observed, 261 and 295 bp alleles had a significant positive association with antibody titres to NDV vaccine at 20 weeks of age when compared to the reference allele (343 bp). However, no alleles were found to be significantly associated with growth, production, NAb titres to RRBC or survivability traits in this breed (Table 5).

In WLH, the 403 bp allele had a significant negative influence on body weight recorded at 16 weeks of age. The negative effect of this allele was also observed on body weight recorded at a different age, but they were not significant. When compared to the reference allele (261 bp), 309 and 552 bp alleles were significantly negatively associated with the egg production up to 72 weeks of age. The 309 bp allele was also significantly negatively associated with survivability of hens up to 72 weeks of age. No allele was found to have significant effect on shank length, age at first egg or immunity traits in this breed (Table 5).

## 4 Discussion

Considering the number of alleles, genotypes and heterozygosity of the LEI0258 VNTR locus, it was evident that genetic diversity at MHC in indigenous breeds was higher than that of the WLH breed. Indigenous breeds were not subjected to artificial selection, and only natural selection was operating in their rearing environment to favour individuals with higher resistance to various diseases that prevailed in the field as MHC is linked with disease resistance, and hence the higher diversity was observed in indigenous breeds. Furthermore, the Ghagus breed was recently collected from its native tract (Six generations back), while WLH was subjected to selection for higher egg production for last several generations, and hence, some degree of inbreeding could have taken place in this breed. Previous studies also reported higher diversity in the MHC-linked LEI0258 locus in indigenous chickens of various countries. A high number of alleles of LEI0258 was reported in eight ecotypes (46 alleles) of Kenya (Ngeno et al., 2014), three indigenous breeds (25 alleles) of Iran (Nikbakht et al., 2013) and in 33 indigenous breeds (69 alleles) of China (Han et al., 2013). On the other hand, lesser genetic variability and diversity at MHC were reported in intensively selected commercial egg layer varieties (Izadi et al., 2011) and commercial white egg layers (Chazara et al., 2013) previously. Higher allelic diversity observed in indigenous chicken breeds can act as a source of unique or novel MHC haplotype types that could be helpful in deciding breeding strategies for conservation and improvement of indigenous chickens for rearing in free-range or backyard systems of production.

The most frequently observed allele was 261 bp among the three breeds. The alleles that were common to the three breeds were 261 and 309 bp. Finding of the high frequency of the 261 bp allele in WLH (66.0 %) and to some extent in Nicobari (22.8 %) breeds as compared to the Ghagus (7.6 %) breed could be explained by the fact that the 261 bp allele is known to be associated with the B15 haplotype (Fulton et al., 2006), and this haplotype is reported to be linked with the high egg production (Lunden et al., 1993). WLH is an egg-type improved breed which was selected for higher egg production, while Nicobari is known to have the highest egg production among indigenous breeds of India. Therefore high frequency of the 261 bp allele was observed in these two breeds.

The present study uncovered 12 unique/novel alleles (248, 272, 274, 284, 346, 354 355, 374, 378, 403 and 565 bp) that were not reported previously either by genotyping or by sequencing (Lima-Rosa et al., 2005; Fulton et al., 2006; Lwelamira et al., 2008; Schou et al., 2010; Chazara et al., 2013; Nikbakht et al., 2013; Han et al., 2013; Guangxin et al., 2014; Esmailnejad et al., 2017; Mwambene et al., 2019). The size of 403 and 565 bp alleles were confirmed by sequencing. However, the size of remaining alleles was determined by extrapolating the genotyped sizes. Therefore, the precise size of these alleles needs to be confirmed by sequencing. Nevertheless, the findings suggest the possibility of indigenous chickens (seven in Ghagus, three in Nicobari and two in WLH) possessing the unique alleles of the LEI0258 marker. Only BW3 (194 bp), 13.1 (381 bp), 19 (539 bp) and 19.1 (552 bp) serologically defined B haplotypes could be correlated among the three breeds. These findings are in conformity with the previous study, which reported that birds of Indian origin had completely unknown haplotypes, and only few serologically defined haplotypes (B2, B12 and B21) could be detected in four lines of indigenous chickens of India (Baelmans et al., 2005), thus reflecting potentially interesting genetic pool in indigenous breeds.

Ghagus and Nicobari breeds deviated from HWE. The deviation from HWE occurs due to selection (natural or artificial), missing genotypes, genetic drift, mutation, migration, population substructure, genotyping error, etc. (Chen et al., 2017). Genotyping error in this study was ruled out as sizes of alleles were first determined on agarose gel electrophoresis, and the precise size of all alleles were then determined on automated analyser, and the procedures used were common to all three breeds. The possible reason for deviation could be missing genotypes, population substructure or natural selection observed in both indigenous breeds of chicken. Similar deviation from HWE was reported with high heterozygosity at MHC in chicken populations originated in China (Izadi et al., 2011). Similar to the findings of our study, Mwambene et al. (2019) also reported significant deviation from HWE for the LEI0258 marker in 6 out of 10 indigenous chicken ecotypes of Africa. It was opined that deficiency of heterozygosity (lesser $H_{o}$ than $H_{e}$) or excess heterozygosity could be one among many reasons for the deviation. In our case perhaps excess heterozygosity observed particularly in Ghagus could be one of the reasons for deviation from HWE. Expected and unbiased expected heterozygosities were similar in all three breeds. Fixation index (F), which indicates degree of inbreeding was the lowest in Ghagus followed by WLH and Nicobari breeds. F statistic (Fst) observed for this LEI0258 marker was 0.172. This suggests that 17.2 % of the genetic variation was due to breed differences, and the rest of the variation was due to differences between individuals within each breed, suggesting significant genetic differentiation among the three breeds (Wright, 1978).

The genetic distance (Nei) between Ghagus and Nicobari breeds was higher as compared to Ghagus and WLH and that between Nicobari and WLH breeds. Mainland India and Andaman and Nicobar islands are separated by Bay of Bengal, and therefore there was least chance for genetic relatedness among these two indigenous breeds. Closer genetic relatedness observed between Nicobari and WLH was somewhat unexpected, and this can be explained by the fact that the Nicobari breed was reported to have originated from the crosses of different exotic breeds such as WLH, Australorp, Plymouth Rock, etc., and indigenous fowl of the Nicobar group of islands (Chatterjee et al., 2005). Therefore, closer genetic relationship was observed between Nicobari and WLH breeds. Therefore, Ghagus was genetically distant from both Nicobari and WLH breeds. Similar to the findings of the present study, Ahlawat et al. (2004) reported the high genetic similarity (0.80) between Nicobari and WLH breeds of chicken using RAPD analysis.

Alleles of LEI0258 had a significant association with body weight, egg production and survivability traits in Ghagus and WLH breeds and antibody titres to NDV in the Nicobari breed. Significant positive association of LEI0258 alleles with body weight at 16 weeks of age was also reported in Tanzanian native chickens (Lwelamira et al., 2008), and negative association with body weight at 8 and 12 weeks of age was reported in Iranian Khorasan native chickens (Nikbakht and Esmailnejad, 2015). Survivability of hens is an important economic trait which has bearing on production performance. A positive association of 194 bp allele with egg production up to 72 weeks of age in Ghagus can be explained from the fact that the survivability of hens with the 194 bp allele was also significantly higher as compared to the reference allele (309 bp). Allele 309 bp, which had negative association with survivability of hens up to 72 weeks of age, also had negative association with egg production up to 72 weeks of age. This might be an indirect association as hens which survive for shorter/longer duration will have lesser/higher chances of producing less/more eggs. Egg production up to 72 weeks of age was highest in hens with the 261 bp allele (reference alleles), which has the highest frequency in the WLH breed. Similarly, the survivability of hens having the 261 bp allele was highest as compared to other alleles (except 403 bp allele) in the WLH breed. Significant association of LEI0258 alleles with the egg laying intensity trait was also reported in Khorasan indigenous chickens of Iran (Nikbakht and Esmailnejad, 2015). Another study reported the significant association of alleles of the BF2 locus of MHC with early and late mortality in commercial broiler line (Ewald et al., 2007) suggesting the role of MHC haplotypes in survivability trait of chickens. However, to our knowledge this is the first study to report the effects of LEI0258 alleles on duration of the survivability of hens during the 72-week laying period.

In Nicobari, LEI0258 alleles (261 and 295 bp) were positively associated with antibody titres to NDV vaccine. Similar to the findings of the present study, significant association of some of the LEI0258 alleles with antibody response to NDV was also reported in Tanzanian chicken ecotypes (Lwelamira et al., 2008) and broiler chickens (Esmailnejad et al., 2017). These findings reiterate the importance of MHC in vaccine response to the NDV antigen. Among viral diseases, incidence of Newcastle disease is the highest in native chickens reared in backyard or free-range systems of production. Therefore, information on diversity at MHC helps in devising breeding strategies for conservation and improvement of native chickens.

Associations between LEI0258 alleles and survivability and immune traits observed in the present study can result from direct effect of MHC genes, which are known to influence disease resistance and immune competence traits. Those of production traits may be due to indirect association between survivability (longer duration of production) and egg production up to 72 weeks of age. Almost all associations of LEI0258 alleles with production traits were found to be breed specific. This is due to the fact that (except for the 261 bp allele) no alleles were found to be common to all three breeds and that reference alleles were different among the three breeds to determine the effect of each allele in comparison to the reference allele. Therefore, allelic effects on various traits need to be studied breed/line-wise (Ewald et al., 2007) and this is particularly relevant in indigenous chicken breeds due to prevalence of high diversity at MHC.

## 5 Conclusions

From the findings of the present study, it is concluded that the LEI0258 marker was highly polymorphic in indigenous breeds, and the number of alleles identified in each breed was different. Considering various parameters examined like number of observed and effective number of alleles, genotypes, heterozygosity, etc., it was evident that indigenous chicken breeds showed higher genetic variability at the MHC region over WLH egg-layer birds. Furthermore, the indigenous breeds likely to have novel alleles of the LEI0258 marker. Some of the alleles were found to have significant association with growth, production, survivability and antibody titres to NDV. Further studies are required to explore the MHC diversity in indigenous chickens through sequencing of the LEI0258 marker for SNP and indel polymorphisms and also to investigate the effects of novel alleles on various immune-competences, disease resistance and production traits using large number of samples particularly in indigenous chicken breeds.

## Data Availability

The data from the paper are available upon request from the corresponding author.

## References

[bib1.bib1] Ahlawat SPS, Sunder J, Kundu A, Chatterjee RN, Rai RB, Kumar B, Senani S, Saha SK, Yadav SP (2004). Use of RAPD-PCR for genetic analysis of Nicobari fowl of Andaman. Brit Poultry Sci.

[bib1.bib2] Baelmans R, Parmentier HK, Nieuwland MG, Dorny P, Demey F (2005). Serological screening for MHC (B)-polymorphism in indigenous chickens. Trop Anim Health Pro.

[bib1.bib3] Banat GR, Tkalcic S, Dzielawa JA, Jackwood MW, Saggese MD, Yates L, Kopulos R, Briles WE, Collisson EW (2013). Association of the chicken MHC B haplotypes with resistance to avian coronavirus. Dev Comp Immunol.

[bib1.bib4] Besbes B (2009). Genotype evaluation and breeding of poultry for performance under sub-optimal village conditions. World Poultry Sci J.

[bib1.bib5] Chatterjee RN, Yadav SP (2008). Farming System of Nicobari Fowl – An Endangered Breed of Andaman and Nicobar Islands, India. World Poultry Sci J.

[bib1.bib6] Chatterjee RN, Yadav SP, Rai RB (2005). Breed descriptor of Nicobari fowl. Bulletin.

[bib1.bib7] Chazara O, Chang C, Bruneau N, Benabdeljelil K, Fotsa J, Kayang BB, Loukou NE, Osei-Amponsah R, Yapi-Gnaore V, Youssao IAK, Chen C, Pinard-van der Laan M, Tixier-Boichard M, Bed'Hom B (2013). Diversity and evolution of the highly polymorphic tandem repeat LEI0258 in the chicken MHC-B region. Immunogenetics.

[bib1.bib8] Chen B, Cole JW, Grond-ginsbach C (2017). Departure from Hardy Weinberg Equilibrium and genotyping error. Front Genet.

[bib1.bib9] Emam M, Mehrabani-yeganeh H, Barjesteh N, Nikbakht G, Thompson-crispi K, Charkhkar S, Mallard B (2014). The influence of genetic background versus commercial breeding programs on chicken immunocompetence. Poultry Sci.

[bib1.bib10] Esmailnejad A, Brujeni GN, Badavam M (2017). LEI0258 microsatellite variability and its association with humoral and cell mediated immune responses in broiler chickens. Mol Immunol.

[bib1.bib11] Ewald SJ, Ye X, Avendano S, McLeod S, Lamont SJ, Dekkers JCM (2007). Associations of BF2 alleles with antibody titres and production traits in commercial pure line broiler chickens. Anim Genet.

[bib1.bib12] Fulton JE, Juul-Madsen HR, Ashwell CM, McCarron AM, Arthur JA., O'Sullivan NP, Taylor Jr RL (2006). Molecular genotype identification of the Gallus gallus major histocompatibility complex. Immunogenetics.

[bib1.bib13] Guangxin E, Sha R, Zeng S, Wang C, Pan J, Han J (2014). Genetic variability, evidence of potential recombinational event and selection of LEI0258 in chicken. Gene.

[bib1.bib14] Han B, Lian L, Qu L, Zheng J, Yang N (2013). Abundant polymorphisms at the microsatellite locus LEI0258 in indigenous chickens. Poultry Sci.

[bib1.bib15] Haunshi S, Burramshetty AK, Kannaki TR, Raja Ravindra KS, Chatterjee RN (2017). Pattern recognition receptor genes expression profiling in indigenous chickens of India and White Leghorn. Poultry Sci.

[bib1.bib16] Haunshi S, Arun Kumar B, Kannaki TR, Rajkumar U (2019). Survivability, immunity, growth and production traits in indigenous and White Leghorn breeds of chicken. Brit Poultry Sci.

[bib1.bib17] Izadi F, Ritland C, Cheng KM (2011). Genetic diversity of the major histocompatibility complex region in commercial and noncommercial chicken flocks using the LEI0258 microsatellite marker. Poultry Sci.

[bib1.bib18] Kannaki TR, Reddy MR, Dhanutha NR, Shanmugam M, Chatterjee RN, Rajkumar U, Haunshi S (2010). Differential expression of Toll-like receptor mRNA in White Leghorn and indigenous chicken of India. Vet Res Commun.

[bib1.bib19] Karnati HK, Pasupuleti SR, Kandi R, Undi RB, Sahu I, Kannaki TR, Subbiah M, Gutti RK (2015). TLR-4 signalling pathway: MyD88 independent pathway up-regulation in chicken breeds upon LPS treatment. Vet Res Commun.

[bib1.bib20] Lima-Rosa CAV, Canal CW, Fallavena PRV, Freitas LB, Salzano FM (2005). LEI0258 microsatellite variability and its relationship to B-F haplotypes in Brazilian (blue-egg Caipira) chickens. Genet Mol Biol.

[bib1.bib21] Lunden A, Edfors-Lilja I, Johansson K, Liljedahl LE, Simonsen M (1993). Associations between major histocompatibility complex genes and production traits in White Leghorns. Poultry Sci.

[bib1.bib22] Lwelamira J, Kifaro GC, Gwakisa PS, Msoffe PLM (2008). Association of LEI0258 microsatellite alleles with antibody response against Newcastle disease virus vaccine and body weight in two Tanzania chicken ecotypes. Afr J Biotech.

[bib1.bib23] Mwambene PL, Kyallo M, Machuka E, Githae D, Pelle R (2019). Genetic diversity of 10 indigenous chicken ecotypes from Southern Highlands of Tanzania based on Major Histocompatibility Complex-linked microsatellite LEI0258 marker typing. Poultry Sci.

[bib1.bib24] McConnell SK, Dawson DA, Wardle A, Burke T (1999). The isolation and mapping of 19 tetranucleotide microsatellite markers in the chicken. Anim Genet.

[bib1.bib25] Miller MM, Taylor Jr RL (2016). Brief review of the chicken Major Histocompatibility Complex: the genes, their distribution on chromosome 16, and their contributions to disease resistance. Poultry Sci.

[bib1.bib26] Nei M (1973). Analysis of gene diversity in subdivided populations. P Natl Acad Sci USA.

[bib1.bib27] Nei M (1978). Estimation of average heterozygosity and genetic distance from a small number of individuals. Genetics.

[bib1.bib28] Ngeno K, Van Der Waaij EH, Megens HJ, Kahi AK, Van Arendonk JAM, Crooijmans RPMA (2014). Genetic diversity of different indigenous chicken ecotypes using highly polymorphic MHC-linked and non-MHC microsatellite markers. Anim Genet Resources.

[bib1.bib29] Nikbakht G, Esmailnejad A (2015). Chicken major histocompatibility complex polymorphism and its association with production traits. Immunogenetics.

[bib1.bib30] Nikbakht G, Esmailnejad A, Barjesteh N (2013). LEI0258 Microsatellite variability in Khorasan, Marandi, and Arian Chickens. Biochem Genet.

[bib1.bib31] Owen JP, Delany ME, Mullens BA (2008). MHC haplotype involvement in avian resistance to an ectoparasite. Immunogenetics.

[bib1.bib32] Peakall R, Smouse PE (2006). GenAlEx 6: Genetic analysis in Excel. Population genetic software for teaching and research. Mol Ecol Notes.

[bib1.bib33] Peakall R, Smouse PE (2012). GenAlEx 6.5: Genetic analysis in Excel. Population genetic software for teaching and research – an update. Bioinformatics.

[bib1.bib34] Rai RB, Ahlawat SPS (1995). Evaluation of disease resistance characteristics of Nicobari fowl. Ind Vet J.

[bib1.bib35] Sambrook J, Russell DW (2001). Molecular cloning: a laboratory manual, 3 Vol.

[bib1.bib36] Schou TW, Labouriau R, Permin A, Christensen JP, Sørensen P, Cu HP, Nguyen VK, Juul-Madsen HR (2010). MHC haplotype and susceptibility to experimental infections (Salmonella Enteritidis, Pasteurella multocida or Ascaridia galli) in a commercial and an indigenous chicken breed. Vet Immunol Immunop.

[bib1.bib37] Sunder J, Chatterjee RN, Rai RB, Ahlawat SPS, Kundu A, Senani S, Saha SK, Yadav SP, Bhagat D (2004). Outbreak of infectious bursal disease in poultry of A and N Islands. Ind Vet Med J.

[bib1.bib38] Suzuki KT, Matsumoto E, Kobayashi E, Matsumoto T, Kobayashi E, Uenishi H, Churkina I, Plastow G, Yamashita H, Hamasima N, Mitsuhashi T (2010). Genotypes of chicken major histocompatibility complex B locus associated with regression of Rous sarcoma virus J-strain tumors. Poultry Sci.

[bib1.bib39] Wright S (1978). Variability within and among natural populations. Evolution and the genetics of populations, Vol 4.

[bib1.bib40] Yadav SP, Kannaki TR, Mahapatra RK, Paswan C, Bhattacharya TK, Sarkar SK, Chatterjee RN (2018). In vivo cell-mediated immune, hemagglutination inhibition response, hematological and biochemical values in native vs. exotic chicken breeds. Poultry Sci.

